# Exhaled Aldehydes and Ketones as Biomarkers of Lung Cancer and Diabetes: Review of Sensor Technologies for Early Disease Diagnosis

**DOI:** 10.3390/bios15100668

**Published:** 2025-10-03

**Authors:** Rafał Kiejzik, Tomasz Wasilewski, Wojciech Kamysz

**Affiliations:** Department of Inorganic Chemistry, Faculty of Pharmacy, Medical University of Gdańsk, Hallera, 107, 80-416 Gdańsk, Poland; rafal.kiejzik@gumed.edu.pl (R.K.);

**Keywords:** breath, aldehydes, ketones, volatile organic compounds, biomarkers, nanomaterials, molecularly imprinted polymers, lung cancer, sensors, biosensors, electronic nose

## Abstract

Exhaled breath (EB) contains numerous volatile organic compounds (VOCs) that can reflect pathological metabolic processes, making breath analysis a promising non-invasive diagnostic approach. In particular, volatile aldehydes and ketones have been identified as disease biomarkers in EB. Gas sensors are expected to play a crucial role in the diagnosis of numerous diseases at an early stage. Among the various available approaches, sensors stand out as especially attractive tools for diagnosing diseases such as lung cancer (LC) and diabetes, due to their affordability and operational simplicity. There is an urgent need in the field of disease detection for the development of affordable, non-invasive, and user-friendly sensors capable of detecting various biomarkers. Devices of the new generation should also demonstrate high repeatability of measurements and extended operational stability of the employed sensors. Due to these demands, the past few years have seen significant advancements in the development and implementation of electronic noses (ENs), which are composed of an array of sensors for the determination of VOCs present in EB. To meet these requirements, the development and integration of advanced receptor coatings on sensor transducers is essential. These coatings include nanostructured materials, molecularly imprinted polymers, and bioreceptors, which collectively enhance selectivity, sensitivity, and operational stability. However, reliable biomarker detection in point-of-care (PoC) mode remains a significant challenge, constrained by several factors. This review provides a comprehensive and critical evaluation of recent studies demonstrating that the detection of VOCs using gas sensor platforms enables disease detection and can be implemented in PoC mode.

## 1. Introduction

In recent years, there has been significant development of gas sensors for detecting VOCs [1,2]. VOCs are molecules that usually have low molecular weights, low boiling points, and high vapor pressures, enabling them to evaporate at room temperature [3]. Aldehydes and ketones are categories belonging to VOCs present in human EB [4]. These compounds are among the most commonly mentioned VOCs as potential biomarkers [5]. The identification of biomarkers, especially in exhaled air, has the potential for clinical application in many lung diseases, including LC, as well as other serious diseases such as asthma and pneumonia [6,7]. Among atmospheric aldehydes, formaldehyde and acetaldehyde are the most commonly occurring [8]. Currently, optimal solutions are being sought for the identification and quantification of VOCs in environmental and food samples, as well as in disease diagnostics. VOCs, including aldehydes and ketones, present in ambient air represent an emerging environmental concern, necessitating systematic monitoring and detection. Currently, analytical techniques such as Gas Chromatography–Mass Spectrometry (GC-MS) are employed for their identification and quantification. However, due to the limitations of GC-MS in routine analytical applications, an innovative approach involves the implementation of EN devices, a technology enabling real-time odor analysis with potential for field deployment [9]. With around 20,000 breaths taken daily by every human, it is easy to see why breath analysis is one of the simplest and most accessible methods for assessing a person’s health [10]. An excessively high concentration of VOCs in EB may serve as a potential biomarker, indicating the presence of disease. Volatile aldehydes have been classified as biomarkers for various diseases, including cancers, and can be detected in EB [11]. However, aldehydes are not the only VOCs present in exhaled air, with over 800 identified [12]. Recent studies have shown that exhaled aldehydes are significantly elevated in lung cancer patients and can discriminate them from healthy controls with high sensitivity, supporting aldehydes as potential biomarkers for lung disease diagnosis [13]. A significant contributor to the high mortality rates caused by multifactorial diseases is frequent diagnosis at advanced stages. Late-stage detection not only reduces the effectiveness of treatment but also significantly increases healthcare costs [14,15]. Currently, intensive research is being conducted to develop novel non-invasive diagnostic methods, focusing on breath analysis of VOCs as key biomarkers that reflect metabolic and biochemical changes in many diseases [16]. Exhaled breath analysis is a promising non-invasive tool for LC diagnosis and monitoring [17,18]. Diabetes is also emerging as one of the next major global health challenges, and early detection is crucial to avoid severe, irreversible complications. Exhaled acetone concentration correlates closely with blood glucose levels in diabetes. Diabetic patients show markedly higher acetone in EB compared to healthy individuals, indicating acetone’s promise as a non-invasive biomarker for diabetes diagnosis and monitoring [19]. Research indicates that elevated levels of VOCs in EB, particularly ketones such as acetone, serve as biomarkers for the metabolic dysregulation associated with diabetes [19]. These discoveries have contributed to intensive research into non-invasive methods of diagnosing diseases through breath analysis. Depending on the compound studied, its concentration in EB typically occurs at ppm/ppb levels or lower and requires the use of advanced analytical techniques for reliable detection and quantification. To illustrate recent progress, some sensor platforms already achieve ppm detection levels. For instance, nano-SnO_2_ powders enabled hexanal detection at 0.05 ppm, and the OBPP4-linker peptide enabled nonanal detection with a LOD as low as 2 ppm.

GC-MS plays a crucial role in the quantification of VOCs in breath samples. Its high sensitivity and specificity make it especially appropriate for applications where accurate detection of trace levels of VOCs is essential [9,17]. There are many reasons why novel methods for analyzing human breath samples for patient diagnostics are needed. Using current devices such as GC-MS for VOC detection and quantification of samples is associated with many inconveniences, including complicated operation requiring trained personnel, expensive equipment, time-consuming sample preparation steps, and a bulky size. These factors prevent the widespread use of breath sample analysis. Currently, particular attention is focused on the development and miniaturization of non-invasive, portable sensors [20]. The goal is to create solutions that offer high sensitivity, low device costs, simplicity of use, and quick analysis. Devices of the new generation should also demonstrate high repeatability of measurements and extended operational stability of the employed sensors [21]. GC-MS also requires periodic calibration and servicing of the equipment. These disadvantages make such techniques difficult to use in diagnostic laboratories and in PoC settings [17]. Other methods are also used to analyze VOCs, such as surface-enhanced Raman spectroscopy (SERS) [7,22,23], proton transfer reaction mass spectrometry (PTR-MS) [24], and selected ion flow tube mass spectrometry (SIFT-MS) [25]. Much attention is also paid to the possibility of analyzing EB by ENs [9,26,27]. In recent years, research has focused on implementing ENs as diagnostic tools [9,26,28] and as instruments for measuring odor concentrations in the environment [29].

**Figure 1:**
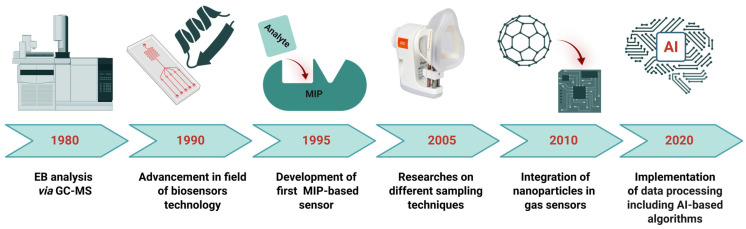
A timeline illustrates the development and advancement in the field of EB analysis, along with critical milestones in sensor development. Created with BioRender.com.

Moreover, the field of gas sensors for LC or diabetes screening is dynamic and continuously evolving (Figure 1), and researchers are working to enhance diagnostic capabilities and establish clinical utility, particularly for disease-specific biomarkers like ketones and aldehydes [30,31,32]. This comprehensive review explores the current state of sensor-based breath analysis for disease diagnosis through the detection of aldehydes and ketones present in EB. It discusses the various sensor types employed, and assesses their advantages, limitations, and translational potential for routine clinical practice and PoC applications. The next section of this paper introduces potential volatile biomarkers in EB and sampling methodologies, with a particular focus on aldehydes and ketones, as well as the current challenges associated with these areas. This is followed by an overview of conventional analytical techniques such as GC-MS and a discussion of sensor technologies, including nanostructured materials, bioreceptors, and molecularly imprinted polymers (MIPs). The final section highlights the key challenges and future prospects for integrating breath analysis into early disease diagnostics. By highlighting the progress and challenges in this field, this review aims to advance EB analysis methodologies and their potential to transform the diagnosis of common diseases.

## 2. Potential Volatile Biomarkers in Exhaled Breath

Invasive diagnosis methods are often associated with varying degrees of patient discomfort, which can discourage participation in routine controls, reduce sampling frequency, and increase overall costs in future treatment [33,34]. Conventional diagnostic techniques, such as blood sampling, tissue biopsy, mammography, gastroscopy, and colonoscopy, are still widely employed today, although they are inherently invasive procedures. These methods often result in patient discomfort and pain, which can contribute to reduced compliance with routine medical screenings [35]. Furthermore, many of these classical diagnostic approaches are both costly and time-consuming, limiting access to regular preventive tests in many parts of the world, especially in third-world or developing countries [36]. Due to the limitations summarized in Table 1 below, considerable research efforts are focused on the development and implementation of non-invasive sampling methods that address most of the problems. Such approaches, including breath analysis, offer a viable alternative.

**Figure 2:**
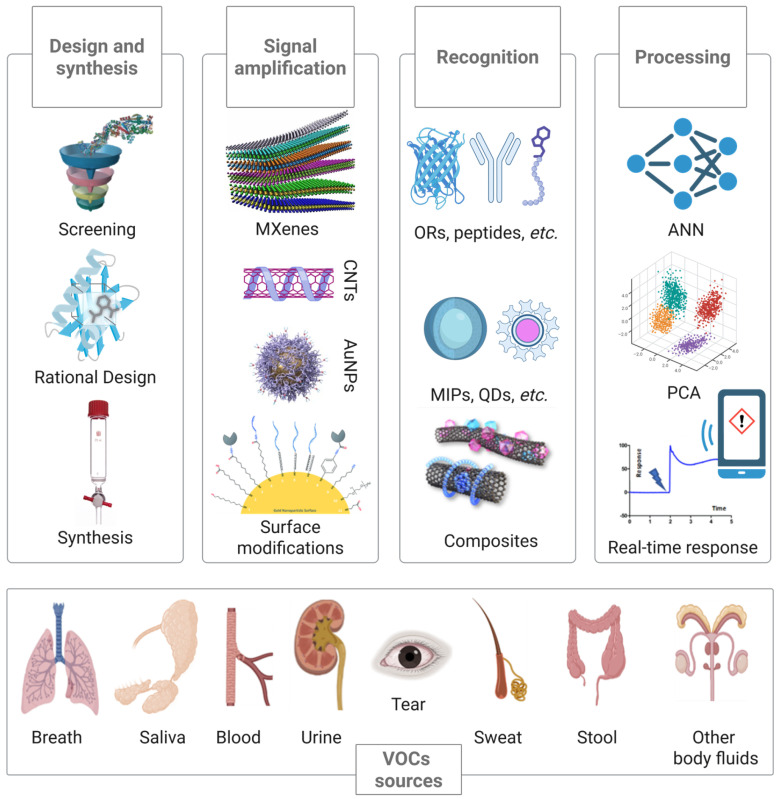
Conceptual schematic of sensor design for VOC detection. The workflow illustrates the main stages: design and synthesis of recognition elements; signal amplification strategies and surface modifications; and data processing and analysis of the obtained signals. The main sources of disease-related volatile biomarkers in the human body are presented below.

Unlike traditional biofluids such as saliva or urine, EB offers a direct, real-time snapshot that often reflects pathological processes in the human body. Each disease is associated with a “fingerprint”, which is a specific pattern of emitted VOCs resulting from pathological metabolic processes. Most of the VOCs in EB are produced in the body through cellular metabolism or oxidative stress [44,45,46]. After being generated, they are released and can be detected in EB. Volatile compounds can be detected not only in EB but also in various other biological matrices, e.g., blood [47], sweat [48], urine [49], and saliva [50,51] (Figure 2). Over the past few decades, thousands of volatile markers associated with various diseases have been identified and categorized from multiple biological sources [52]. VOCs, particularly aldehydes and ketones [13,45], have gained increasing attention as potential biomarkers associated with various pathological conditions and can be analyzed by several methods, including sensors (Figure 2) [7,53,54]. Among the most promising platforms are electrochemical sensors [55], fluorescence-based sensors [56], and physical sensors (e.g., colorimetric or chemiresistive) [57]. Their presence and concentration in EB offer a promising, non-invasive approach for early disease detection and clinical monitoring. Diagnosing diseases presents several challenges, and the detection of volatile biomarkers has become increasingly important due to the non-invasive nature of breath analysis and the possibility of quickly collecting a sample of exhaled air. The subsequent section provides an overview of current EB sampling methodologies, which constitute a critical prerequisite for reliable biomarker detection.

### 2.1. Sampling Methodologies for Exhaled Air

In exhaled air VOC analysis, the initial sampling stage is both critical and demanding, as it directly influences the reliable identification and quantitative determination of volatile biomarkers. A variety of specialized collection systems have been developed to ensure controlled, reproducible sampling, each designed to address key challenges. EB samples are typically collected under controlled conditions and stored in chemically inert containers, such as Tedlar^®^ (SKC Inc., Houston, TX, USA) bags or sorption tubes, to preserve the integrity of VOCs prior to analytical processing [58,59,60]. Currently, Tedlar^®^ bags are one of the most popular and widely accepted tools for gas sampling. This is due to their low price, chemical inertness, relatively good durability, and the capability of multiple uses.

However, the use of Tedlar^®^ bags has certain limitations. Studies have demonstrated analyte losses of less than 20% after 10 h of storage, prompting the recommendation that samples be analyzed within 10 h of collection [58,61]. It has also been shown that storing breath samples for too long can increase the amount of contaminants in the bags, supporting the theory of compound diffusion [58,61,62].

Recently, devices like the Mistral Sampler and ReCIVA^®^ (Owlstone Medical, Cambridge, UK) Breath Sampler have been developed and commercialized, allowing for direct collection and preconcentration of VOCs on sorbent tubes [63,64]. The Mistral system enables simultaneous gas sampling into two independent sorbent tubes. The ReCIVA^®^ Breath Sampler includes four independent adsorbent tubes for VOC collection. A detailed comparison of breath sampling methods has already been conducted and documented in several studies, highlighting the advantages and limitations of the Mistral Sampler and ReCIVA^®^ Breath Sampler [65].

Direct EB analysis is possible with the SICRIT^®^ Breath Analysis Module. This module can be coupled to any mass spectrometer. The company named Plasmion GmbH (Augsburg, Germany) patented a soft ionization source in combination with a heated EB sampling system, which ensures the highest inertness and enables real-time analytical capabilities without sample pre-processing. Sorbent tubes are also employed to preserve and supply collected breath samples [66].

**Figure 3:**
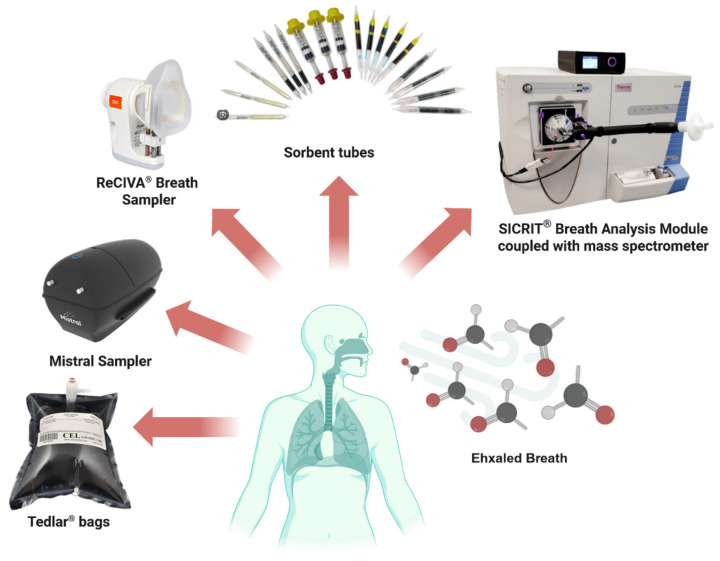
A comparative schematic with currently available EB sampling devices and a device enabling direct analysis of EB with GC/MS.

Furthermore, sorbent tubes packed with Tenax^®^ (Baltimore, MD, USA) are capable of storing asthma-related VOCs for periods of up to two weeks [67]. A schematic overview of EB sampling methods and a device that enables direct analysis of EB samples with GC/MS is presented below (Figure 3). Following the discussion of sampling methodologies, the focus shifts to aldehydes and ketones. These compounds represent the most thoroughly investigated classes of EB biomarkers and currently hold the most promise for clinical diagnostics.

### 2.2. Aldehydes and Ketones as Key Biomarkers Present in Exhaled Breath

In recent years, aldehydes and ketones have garnered significant attention as VOC biomarkers in the non-invasive diagnosis of various diseases. Elevated concentrations of ketones and aldehydes in EB, as detected by EN technology or by GC-MS, may serve as non-invasive biomarkers for pathological states. These compounds, often present in EB, reflect underlying metabolic and oxidative stress processes associated with pathological conditions [46]. The discovery and identification of reliable disease-specific VOCs at detectable concentrations depend on reproducible and accurate analytical methods for gas analysis. Early, quick, and precise diagnosis of patient conditions, including respiratory diseases, as well as timely therapeutic intervention, significantly influence public health outcomes and the efficiency of clinical trials [68].

Acetone is generated by hepatic ketogenesis (β-oxidation of fatty acids) under insulin-deficient conditions [69]. Long-chain aldehydes like octanal and nonanal are produced through the lipid peroxidation of fatty acids in cell membranes, a process that is enhanced by chronic inflammation in LC [45]. Formaldehyde is produced endogenously by oxidative demethylation processes, and its breath level rises under oxidative stress [70,71]. The compilation of ketones and aldehydes with potential diagnostic relevance to a range of diseases is listed below in Table 2. Recent studies summarized in the table provide evidence that the analysis of aldehydes [13,18,72] and ketones [73] in EB can serve as an effective tool for the early detection of various diseases, including LC and diabetes. The widespread adoption of this non-invasive method of breath analysis may contribute to improved patient comfort and also enable frequent monitoring of health status.

Increasing evidence for breath analysis as a diagnostic tool highlights the necessity of characterizing disease-specific VOCs. Therefore, the development of VOC detection technologies, such as chemical and biochemical sensors, should be considered as a key research focus within medical diagnostics. The analysis of VOCs in EB offers numerous advantages in disease detection, therapy planning, and monitoring. Lung cancer remains the leading cause of cancer cases and deaths globally [89]. The net five-year survival rate for LC ranges between 10 and 20% in most countries [90]. However, when detected early, LC is curable with proper treatment. Diabetes represents a growing global health threat, with rising incidence rates and serious complications in the long term. Although significant progress has been made in diabetes management, timely diagnosis is still essential to avoid permanent complications. In this context, the assessment of VOCs in EB emerges as a valuable non-invasive method for both early detection and long-term monitoring of individual patients with diabetes. A meta-analysis noted a strong correlation between breath acetone and blood glucose [91]. To support diagnosis and tracking, it is essential to develop ENs based on sensor platforms with selective sensitivity to long-chain aldehydes such as octanal and nonanal, which are compounds identified as biomarkers of LC [13]. Conducting research on ENs is essential, as their compact design holds significant potential for the development of PoC diagnostic tools [92]. To provide a comprehensive perspective, the final part of this section is dedicated to the key methodological challenges and limitations of EB biomarker research in its transition towards clinical application.

### 2.3. Current Limitations and Challenges in Disease Diagnosis Via Exhaled VOCs

Analytical tools suitable for PoC applications require particular consideration, as many patients lack consistent access to centralized diagnostic facilities. Eliminating the need for sample transportation to specialized laboratories staffed by qualified personnel reduces both time and costs associated with analysis. During epidemics, the absence of simple and reliable detection systems like EN may result in delayed or inaccurate diagnoses, facilitating uncontrolled disease spread [93]. To date, diagnostic methods based on detecting VOCs have not been implemented into routine clinical practice, primarily due to the unresolved methodological and standardization issues outlined below, which influence their reliability and widespread applicability. Despite the growing interest in using EB analysis as a non-invasive diagnostic tool, a lot of challenges and limitations persist in this field. One of the primary constraints is the extremely low concentration of VOCs, such as aldehydes and ketones, in EB, often in the parts-per-million (ppm) or parts-per-billion (ppb) range [94,95] or lower (Table 3). It requires the use of extremely sensitive and selective detection methods that allow the identification of trace amounts of volatile biomarkers.

Another critical barrier is that the exhaled VOC profiles are significantly impacted by endogenous and exogenous confounders, such as age, gender, diet, ethnicity, environmental exposures, and lifestyle, which can change the disease-specific “fingerprint” and complicate data interpretation [98,99,100]. Smoking is also a significant factor affecting breath composition and has been widely studied [60,101]. In addition, the absence of consensus on data preprocessing, together with the need for rigorous validation and standardized reporting of both analytical and statistical methods, makes it difficult to integrate breath analysis into clinically useful diagnostic tools [100]. Another barrier is the lack of standardized protocols for breath sample collection and processing. Variables like sampling device (e.g., Tedlar^®^ bags and sorbent tubes), flow rate, and length of storage can affect VOC quantification and may lead to poor repeatability across multiple studies [58,59]. The design of standardized breath sampling protocols is critical for optimizing exhaled VOC diagnostic methods, as variations in collection procedures may impact the final result.

## 3. Conventional Approaches for VOCs Analysis

Various technologies are being actively developed and implemented for diagnostic applications, particularly for the early detection of diseases. Electronic sensing devices used for complex VOC analysis in gaseous clinical samples offer significant advantages, most notably their capacity for non-invasive early-stage disease identification [27,102]. The selective detection of exhaled aldehydes and ketones (e.g., acetaldehyde, acetone, and nonanal), which are biomarkers associated with COVID-19, could enable faster, non-invasive diagnostics [81,82]. The emphasis on factors such as fast response time, selectivity, high sensitivity, miniaturization capability, and reasonable cost has driven the development of numerous sensors, which represent promising tools due to their compatibility with PoC mode operation. Table 4 presents an overview of the most relevant analytical techniques employed in EB analysis.

GC–MS is the gold standard for analysis of VOCs in EB, including aldehydes and ketones, where the limit of detection LOD oscillates at the ppb level [103]. Nearly all clinical breathomic studies, like those involving lung cancer, have used GC–MS to identify biomarkers [104]. For instance, breath GC–MS surveys have repeatedly found elevated ketones (e.g., acetone) and aldehydes (e.g., hexanal, heptanal, octanal, nonanal, etc.) in LC patients compared to controls [104]. Likewise, GC–MS quantification of acetone from EB has been used in diabetes research. A meta-analysis noted a strong correlation between breath acetone and blood glucose [91,105]. In a large-scale study, the mean breath acetone concentration was 1.5 ± 0.5 ppm in individuals with T2D compared to 1.0 ± 0.6 ppm in healthy subjects, demonstrating that GC–MS can effectively distinguish between diabetic and healthy control breath samples [77]. Despite its accuracy, GC–MS has a lot of disadvantages. The instrumentation is bulky, requiring trained operators, standardization, and time-consuming sample preparation [106]. With GC–MS, analysis of acetone in EB requires sample preparation [69,107]. Due to these delays and the need for preconcentration, GC–MS is incapable of providing real-time results. Additionally, GC–MS workflows are generally costly and time-consuming.

## 4. Gas Sensors for the Detection of Aldehydes and Ketones Present in EB

**Figure 4:**
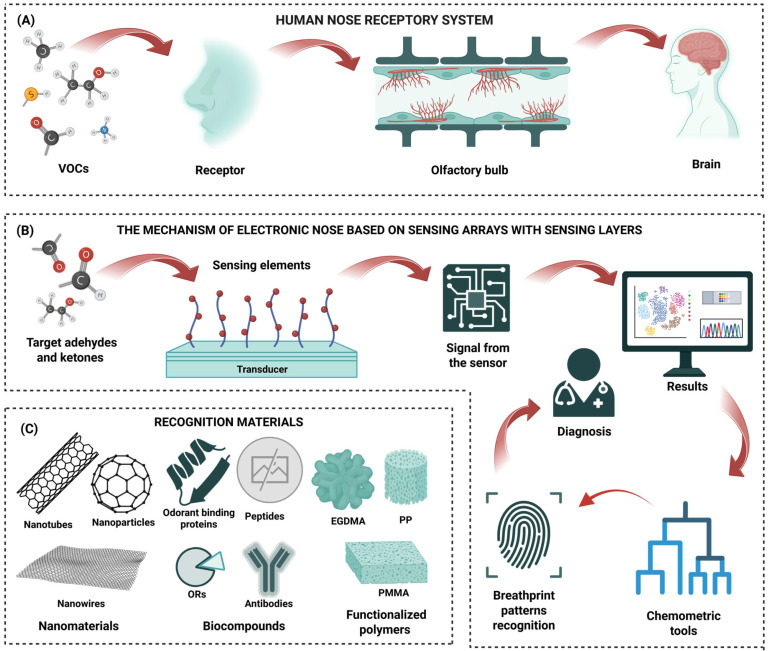
(**A**) Comparison of the mechanism of the human nose based on olfactory receptor protein, inspiring the concept of an EN device designed to mimic the olfactory mechanism of humans [115]. (**B**) A highly selective sensor array for aldehydes and ketones, and the sensor array’s working mechanism. (**C**) Examples of recognition materials are discussed in the next subsection.

In the current clinical conditions, it is extremely important to develop a novel method for the quick detection and monitoring of VOCs for diagnostic purposes. Faster diagnosis can make treatment easier and even save a patient’s life. Therefore, it is crucial to create a device that allows for fast analysis of EB samples. The detection of aldehydes and ketones is of increasing interest due to their role as biomarkers in non-invasive disease diagnostics. Gas sensors offer a promising alternative to conventional analytical techniques like GC-MS, which is the gold standard in this field [9,17,24,97]. Despite the many advantages of GC-MS, unfortunately, many factors mentioned in the introduction chapter prevent its widespread clinical use, which has led to the search for other diagnostic methods. The field of gas sensing has witnessed lightning technological advancements driven by its pivotal role in environmental monitoring, industrial safety, and biomedical diagnostics. The EB analysis tool should work by replicating the sense of smell, as demonstrated by the olfactory and neurological functions in humans, as clearly illustrated in Figure 4A. Gas sensors are analytical devices that detect and quantify VOCs by converting gas concentrations into measurable electrical, optical, or electrochemical signals, which are directly proportional to the target analyte. Mechanisms of EN devices are designed to mimic the olfactory mechanism of humans, as illustrated in Figure 4B.

In recent years, significant efforts have been directed toward developing sensor platforms specifically optimized for the selective detection of aldehydes, owing to their importance as volatile biomarkers and environmental pollutants. To enable accurate detection of diseases based on analysis of EB, especially considering its complex chemical profile, it is essential to integrate multiple selective sensors into a sensing array [108]. To achieve this goal, many transduction mechanisms have been investigated, including metal oxide semiconductor (MOS) sensors [109,110], fluorescent chemosensors [56,111], electrochemical sensors [72], field-effect transistors (FETs) [112], and piezoelectric sensors [113,114]. Transducers exhibiting interface compatibility can be integrated into EN devices to enhance analytical performance. The use of a transducer platform functionalized with appropriately selected sensing layers enables higher selectivity and sensitivity. Figure 4C illustrates the gas sensing mechanism incorporating commonly employed transducers and recognition layers discussed in this review.

### 4.1. Electronic Nose Devices

The goal is to create technological solutions that offer high sensitivity, low device costs, simplicity of use, and quick analysis. Devices of the new generation should also demonstrate high repeatability of measurements and extended operational stability of the employed sensors. This makes them highly suitable for real-time monitoring and immediate clinical decision-making [71,116]. A portable EN has been effectively applied to detect diabetes through breath analysis, as demonstrated by its ability to identify the elevated acetone “breathprint”; it is an example of using EN technology with 94% accuracy for diabetes classification [71]. A recent systematic review including 4,483 patients assessed the diagnostic performance of EN technology for LC detection. The meta-analysis reported a pooled sensitivity of 0.90 and a specificity of 0.89, indicating clinical potential of using EN [116].

Numerous analytical approaches have been designed to overcome the challenges associated with gas sample analysis. Firstly, the MOS sensors are the most commonly used sensor type in ENs [117,118]. Owing to their number of advantages, MOS sensors represent the predominant sensor technology employed in EN [119]. On the other hand, SAW sensors are widely used piezoelectric sensors applied in ENs [120,121]. SAW sensors use the conversion of electrical and mechanical energy to generate waves via piezoelectric materials. Several piezoelectric transducer technologies, including quartz crystal microbalance (QCM), were integrated within the EN devices to detect and analyze VOCs. A QCM is a piezoelectric resonator that oscillates at a well-defined fundamental frequency. When mass is deposited on its surface, the frequency decreases proportionally. This relationship is quantitatively described by the Sauerbrey equation, first derived in 1959 [122], and the theoretical frequency shift can be calculated according to the Sauerbrey equation, which relates mass loading to frequency shift.

A wide range of commercially available EN systems has been developed for the detection and analysis of VOCs across diverse biomedical, environmental, and industrial applications. These devices use different sensor technologies, signal transduction mechanisms, and pattern recognition strategies, which directly influence their sensitivity, selectivity, and suitability for specific analytical tasks. Table 5 provides an overview of selected branded ENs, highlighting the types of sensing technologies employed in each case.

Despite numerous advantages, this approach is not without limitations. Fundamental challenges of ENs are their reliance on pattern recognition rather than the identification of specific individual molecules. ENs exhibit sensitivity to environmental factors such as humidity, necessitating rigorous calibration procedures and standardized breath sampling protocols to ensure measurement reliability. The technologies mentioned above allow for the analysis of breath samples, but none alone fully meet the needs for portable, selective, and sensitive monitoring of specific biomarkers. This gap motivates the development of selective gas sensor approaches for real-time detection of aldehydes and ketones in EB. Selectivity of the transducers mentioned above is provided by dedicated receptor recognition layers or materials, which mediate specific interactions with target biomarkers, such as aldehydes and ketones. Coating sensor arrays with selected materials enables them to distinguish complex gas mixtures, providing higher selectivity and sensitivity compared to single sensors. These recognition materials include functionalized polymers such as MIPs, biomolecules, or nanostructured materials, which are described in the following sections.

### 4.2. The Role of Nanostructured Recognition Materials

To date, several technologies have been considered for the detection of VOCs by nanostructured recognition materials, including nanosheets, nanowires, and nanospheres [129,130,131,132,133]. The characteristic scale size range (1–100 nm) of nanomaterials results in a significant increase in their surface-to-volume ratio and the interaction sites [134,135]. Additionally, surface modification through the incorporation of specific additives constitutes an alternative approach to improve sensors’ performance; these modifications may provide enhanced selectivity, sensitivity, and quicker time response.

In gas sensors for the analysis of EB, nanoparticles (NPs) are widely investigated. To achieve higher sensitivity and selectivity, metallic NPs are frequently combined with nanomaterials such as carbon nanotubes, graphene, or MOS, forming advanced composites [136]. NPs deposited on sensor surfaces enhance overall sensitivity by creating preferential adsorption sites via defect formation [98]. Reducing NPs’ size maximizes surface area, thereby increasing defect density and ultimately enhancing sensor sensitivity [137]. A contribution within the current scientific research on NPs-based sensing platforms is presented by Peng et al. [138], who engineered a chemiresistive sensor array employing gold nanoparticles (Au NPs, 5–10 nm) functionalized with diverse organic ligands to detect exhaled VOCs for LC diagnosis. Surface functionalization critically enhanced VOC selectivity, robustness, and fabrication of this sensor array. That approach demonstrates significant potential for non-invasive LC detection and potentially improving patient outcomes, providing a foundation for future advancements. Likewise, Zhao et al. [139] engineered a chemiresistor array using molecularly linked Au NPs, capable of detecting VOC mixtures and LC breath biomarkers with an acetone LOD of 20 ppb. On the other hand, SnO_2_ nanosheets have been developed to selectively detect nonanal in gaseous samples, which is one of the biomarkers of LC [140,141].

Consequently, recent research prioritizes developing MOS sensors based on nanostructured recognition materials specifically designed for these compounds. Acetone sensors can be used for diabetes screening, while sensors sensitive to acetaldehyde and long-chain aldehydes are targeted for early LC detection [77,142]. Table 6 compiles representative nanostructured materials used in MOS sensors designed for the selective detection of key volatile aldehydes and ketones in EB. These sensors include diverse material architectures and mixed-oxide nanocomposites, and incorporate tailored catalytic functionalization. They operate under optimized thermal conditions to maximize sensitivity, selectivity, and stability in the analysis of biomarkers present in EB.

MOS sensors are fundamental components of EN systems for EB analysis for high-sensitivity detection of VOCs [71]. Numerous advantages of MOS sensors based on nanostructured materials make them well-suited for integration with EN devices, compared to various other sensing approaches. On the other hand, one of the most frequently cited MOS disadvantages in the literature is their requirement for high operating temperatures (250–450 °C) [149], which is achieved through the deliberate selection and material engineering of nanostructures exhibiting a high affinity toward specific compounds. Continuous development of sensor arrays based on nanoscale materials is enabling the practical implementation of quantitative EB screening for diabetes, LC, and other diseases. The determination of aldehydes and ketones, including acetone, hexanal, nonanal, and acetaldehyde in EB (often at ppm–ppb levels), presents significant challenges, yet it is achievable through sensors based on nanostructured materials.

### 4.3. Bioreceptors

**Figure 5:**
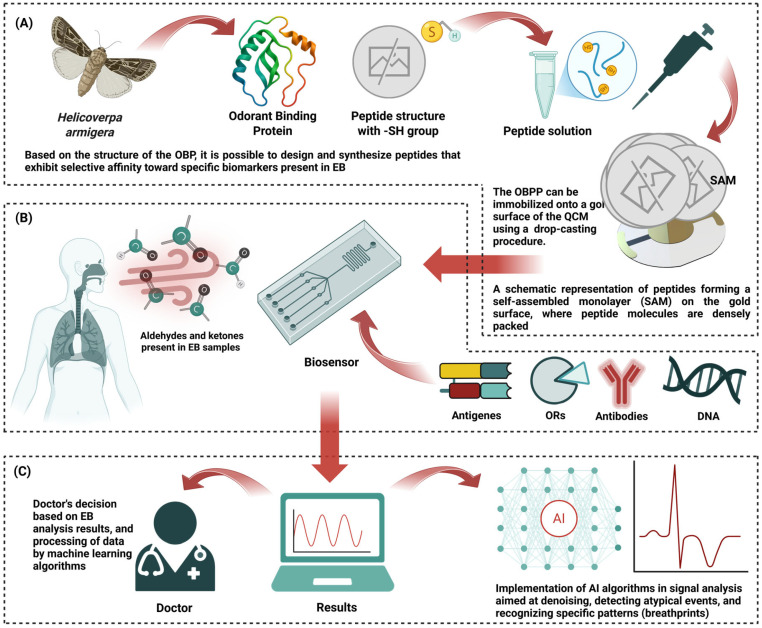
(**A**) This scheme describes the procedure for designing protein/peptide-based biosensors and their deposition on a QCM transducer using the drop-casting method, described in the literature as a quick, low-cost, and sufficiently effective method [154]. (**B**) A biosensor constructed according to this description shows extremely high selectivity and specificity toward aldehydes present in the analyzed EB. The image also highlights other molecules commonly used as bioreceptors. (**C**) The integration of sensor technologies and artificial intelligence systems makes it easier to automate the processing of voluminous results data, significantly enhancing precision. Machine learning, particularly deep neural networks, can detect subtle variations in VOC profiles that are not detectable using traditional data processing methods [155].

One of the other promising approaches in this field of gas sensors involves the use of sensors integrating biological recognition elements that offer enhanced specificity and sensitivity analysis of EB [20,150]. Gas sensors are increasingly using biological receptors, such as olfactory proteins (ORs), odorant binding proteins (OBPs), antibodies, antigens, DNA, or peptides as their selective layers [151,152]. These biosensors mimic the characteristics of animal olfaction and can convert information about the concentration of target odor into a measurable signal. Bioreceptor molecules can be integrated with piezoelectric transducers to enhance target specificity. When immobilized on a QCM, such bioreceptors enable high specificity, sensitivity, and real-time sensing of VOCs [153]. Biosensors based on peptides can be covalently immobilized, often via a terminal cysteine with thiol functional group (-SH), onto the gold surface of QCM, forming a sensing layer (Figure 5A). These biological layers achieve high recognition specificity while preserving sensitivity (Figure 5B). The output results obtained via artificial intelligence (AI) processing can be evaluated and interpreted by a doctor to support clinical decision-making (Figure 5C).

In recent years, studies have been conducted on a large number of OBPs that may find application in EN devices. However, OBP-derived peptides (OBPPs) demonstrate superior functional parameters, including enhanced bioreceptor stability and improved measurement reproducibility, compared to native OBPs. To design such peptides, a comprehensive understanding of the structure of OBPs is required, including the detailed characterization of their binding pockets and analysis of their primary amino acid sequences. Wasilewski et al. designed a peptide with the following sequence: KLLFDSLTDLKKKMSEC (OBPP4), based on a motif identified within HarmOBP7 [156,157]. This protein, previously characterized as a pheromone-binding protein expressed in the antennae of the *Helicoverpa armigera* [158], served as the structural motif.

An alternative approach for the detection of VOCs is the development of biomolecular recognition elements that utilize fluorescence signals in the presence of analytes, like aldehydes and ketones. These compounds are prominent biomarkers for several diseases, including LC and diabetes [11,18,159]. Among the various fluorescent methods [160,161], fluorescent proteins [162] have been employed as a sensing material. For instance, Ye et al. constructed a fiber-optic biochemical gas sensing system by attaching a flow cell with a nicotinamide adenine dinucleotide (NADH)-dependent secondary alcohol dehydrogenase (S-ADH)-immobilized membrane onto a fiber-optic measurement system [163]. The measurement system utilized an ultraviolet light-emitting diode. In Table 7, examples of biosensors developed in recent years are presented, based on either fluorescence or the piezoelectric effect. These biosensors demonstrate high specificity, sensitivity, and the capability for real-time detection of biomarkers present in EB.

### 4.4. Molecularly Imprinted Polymers

However, natural recognition elements have significant limitations for VOC detection. Biomolecules exhibit instability beyond narrow environmental parameters, are susceptible to denaturation, and have limited reusability due to irreversible contamination or degradation [167]. EN devices used in breath analysis are exposed to changing temperature and humidity; therefore, the sensors used in these devices must be resistant to these factors. This instability of biological components constitutes a fundamental constraint. To overcome these challenges, a new approach employs MIPs as the sensing coated layer instead of peptides, proteins, or other biomolecules. MIPs, frequently described as “artificial antibodies”, feature molecular cavities with geometrically precise functional group arrangements that exhibit complementary binding affinity toward target molecules like VOCs [168,169]. Recent years have seen a significant rise in scientific publications focusing on MIPs, with particular attention to their applications in biomarker detection. This upward trend in research output highlights both the growing scientific interest in these sensors and their role as a specialized solution to current analytical challenges. In previous years, MIPs have gained significant attention as highly stable, selective, and cost-effective alternatives to biosensing elements [170]. MIPs are synthesized by copolymerizing monomers and cross-linkers in the presence of target molecules, which are later removed to create specific cavities that are complementary in shape and size [171]. This strategy allows MIPs to mimic the high specificity of biological compounds mentioned in the previous chapter. These sensors have shown great potential as reliable diagnostic tools for breath analysis, effectively solving current clinical problems [98,172]. A summary of the selected MIPs used for the detection of VOCs is presented in Table 8 and Table 9. Below are key examples from recent studies, including analyte biomarkers, monomers, and polymerization methods, as well as the capabilities of several MIP-based sensors. These examples in the following table demonstrate the potential of developing MIPs for the sensitive detection of VOCs.

### 4.5. Overview of Functional Materials as Sensors for EB Analysis

In summary, exhaled breath contains a wide spectrum of VOCs, among which aldehydes and ketones are the most relevant biomarkers for non-invasive diagnostics. To address this challenge, various functional materials have been explored as sensing layers in different transducer systems.

Nanostructured materials, particularly MOS-based sensors, demonstrate high sensitivity through engineered surface modifications, making them promising for detecting acetone and long-chain aldehydes. Bioreceptors, such as peptides and odorant-binding proteins, provide remarkable specificity, although their stability under variable environmental conditions, like humidity, still limits their practical application. In contrast, MIPs offer high chemical and thermal stability, as well as tolerance to changes in humidity.

There is one critical aspect in evaluating the applicability of sensor technologies for breath analysis, which is the comparison between their analytical performance and the actual concentrations of volatile biomarkers present in EB. Clinical studies consistently demonstrate that aldehydes and ketones occur at very low levels, depending on the compound and disease state. This review highlights that several approaches, like nanostructured MOS sensors and MIP-based sensors, already operate within diagnostically relevant ranges; other technologies, such as peptide-based QCM biosensors, still have detection limits far above clinically important concentrations. Therefore, comparing biomarker levels with sensor capabilities offers a clearer view of the field and points to where further sensitivity improvements are most needed for clinical use.

## 5. Challenges and Future Prospects

Despite decades of advancement, the practical use of sensors and biosensors in disease diagnostics remains at an early stage. Current limitations and future directions in the field of sensor-based breath analysis for disease diagnosis are discussed in this section. Sensors for detecting VOCs, especially ketones and aldehydes, require substantial improvements to achieve the precision needed for diagnostic applications. In recent years, sensor technology has been enhanced greatly by the rapid development of sensor arrays, commonly called electronic noses [181]. Key criteria for diagnostic devices, including EN devices, encompass affordability, high sensitivity and specificity, user friendliness, fast response, and wide accessibility [182]. The practical implementation of breath sensors in PoC applications is still limited by several barriers.

One of the most significant is the impact of humidity, which strongly interferes with sensor response, especially for MOS and colorimetric sensors. Water vapor competes with VOCs and can reduce sensitivity, as highlighted in comparative studies of sensor operation under dry and humid environments by Akamatsu et al. [183]. Secondly, there is a study that demonstrates, in line with previous reports, that individual differences can affect the reproducibility and accuracy of results [184,185]. This variability complicates the diagnostic use of breath analysis, as it makes it difficult to establish clear values for key biomarkers such as aldehydes and ketones, which are relevant to LC and diabetes. Furthermore, navigating regulatory and clinical validation results in further significant delays: to gain approval, sensors must meet standards for safety, reproducibility, and accuracy through large, costly, and time-consuming clinical trials [185,186,187].

**Figure 6:**
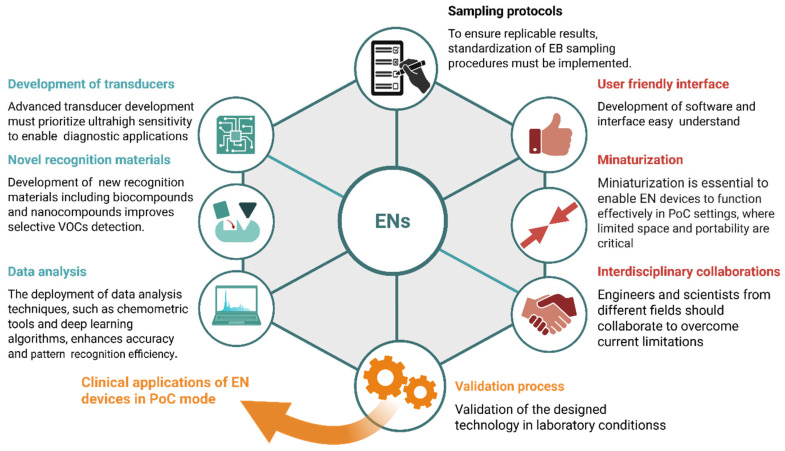
Strategic steps for enhancement of EB analysis for clinical diagnostics through ENs.

EN devices have promising advantages but also several disadvantages mentioned above, and still require key improvements to overcome limitations for medical diagnostic use. The key steps that can be taken to increase the effectiveness of ENs are presented in Figure 6.

Crucially, collaboration among scientists and engineers from diverse disciplines will make progress faster in EB analysis for diagnostic applications in clinical settings. Resolving sensor reliability issues should enhance measurement accuracy. Introducing novel materials, such as polymer composites and nanostructured metals/metal oxides, may improve long-term stability. On the other hand, sensors prioritize selectivity over stability. Meanwhile, research on transducers compatible with sensor arrays and integrable with the recognition layers materials discussed earlier is also important. It is also crucial to ensure that the technology employed remains technically accessible and has a user-friendly interface, enabling EN deployment in PoC settings.

Moreover, integrating advanced data and pattern analytics with AI and chemometric tools can overcome limitations in distinguishing signals from diverse breath biomarkers [188]. Machine learning algorithms (ML) serve as critical analytical tools for pattern detection, enabling the identification of subtle alterations in variable VOC biomarker signatures that correlate with specific disease states [155,169]. Recent studies highlight that integrating ML and AI with sensor arrays and ENs to distinguish individuals with LC from healthy controls based on VOC profiles in EB [189]. This is exemplified by a breath analysis system using a sensor array and deep learning, which achieved a high accuracy of 97.8% in early LC detection, demonstrating the promise of pattern-recognition approaches in improving diagnostic precision of this technology [190]. The integration of AI and ML with digital health platforms would enable predictive analysis and personalized healthcare with high accuracy.

Integration of breath analysis platforms from laboratory development into routine clinical practice requires rigorous clinical validation and regulatory approval. Executing comprehensive clinical studies to evaluate sensor performance parameters, such as sensitivity, specificity, and accuracy, involves significant logistical and financial commitments [16]. Ethical and privacy considerations further complicate deployment. The collection of detailed breath profiles constitutes personal health data subject to protection under regulations. Moreover, transparency in algorithmic decision-making processes and the minimization of potential errors in ML models are essential to uphold ethical standards. Addressing these intertwined clinical, regulatory, ethical, and privacy challenges is crucial for the responsible integration of EN devices into PoC diagnostics. Although technological barriers, such as analyte dilution, biorecognition stability, and sampling standardization, remain unresolved, ongoing advances in sensor engineering and data integration indicate a strong translational potential [191]. With further clinical validation and protocol harmonization, EB sensors may soon emerge as a scalable tool for personalized and preventive medicine.

## 6. Conclusions

This review emphasizes the diagnostic capabilities of gas sensors for clinical applications. Breath analysis may enable earlier detection of diseases such as LC and diabetes in earlier stages. In recent years, there has been significant progress in the development of sensing platforms selective for VOCs (including aldehydes and ketones) present in EB. Nevertheless, despite their promising potential, the innovative sensors discussed above still require further optimization, as VOCs in human EB are present at extremely low concentrations. Endogenous and exogenous factors, including diet, smoking, and environmental exposures, contribute to changes in breath composition and complicate the identification of disease breathprints. Standardized protocols for breath collection and data analysis remain underdeveloped, reducing reliability. Immediate implementation of standardized sampling procedures and protocols is essential for clinical translation. Future research in the field of gas sensor platforms for disease diagnosis via EB should prioritize advanced receptor coatings to achieve higher sensitivity and selectivity. In particular, nanostructured materials or MIPs can be integrated as sensor coatings on various transducers. These materials are easily fabricated and easily integrated with existing sensing platforms, making them suitable for the next generation of ENs. With the advancement of sensor arrays and complex diagnostic data, big data analytics via artificial intelligence will be key enablers of developing novel PoC devices for the analysis of EB. Sensor arrays coupled with AI systems, which facilitate efficient processing of data, could enable the detection of disease from complex VOC signatures. Pattern recognition based on ML algorithms instead of classical data processing methods, and wearable PoC sensor design promise a new era in clinical gas sensing, where biomarkers can be monitored continuously and screening performed widely in hospitals and also in home settings. Successful implementation may yield compact diagnostic devices for non-invasive screening of pulmonary, metabolic, and other diseases. In summary, the detection of VOCs, particularly aldehydes and ketones, may hold promise for early disease detection, provided that existing technical barriers are overcome.

## Figures and Tables

**Table 1:** Comparative summary of prevalent clinical diagnostic methods.

Diagnostic Method	Advantage	Disadvantage	Refs.
Blood sampling	Venipuncture is a minimally invasive, rapid, and cost-effective method for acquiring diagnostic samples, widely used in routine clinical testing.	Even routine phlebotomy can cause patient discomfort and is associated with local complications, such as hematoma formation.	[37,38]
Tissue biopsy	Tissue biopsy provides a source of fresh tumor material for direct histopathological and molecular analyses and remains the gold-standard diagnostic method in clinical oncology.	An invasive procedure, it carries inherent risks including bleeding, infection, and patient discomfort, which limit its repeatability in clinical practice. The procedure requires trained personnel.	[39]
Mammography	Reduces breast-cancer mortality through early detection of asymptomatic lesions.	Employs low-dose ionizing radiation and is associated with a relatively high false-positive rate, resulting in overdiagnosis and subsequent unnecessary follow-up diagnostic tests.	[40,41]
Gastroscopy	Provides direct visualization of the upper gastrointestinal tract while permitting simultaneous tissue sampling via biopsy.	Associated with significant patient discomfort and anxiety. The procedure requires trained personnel.	[42]
Breath sampling	Completely non-invasive and quick method, offering potential for PoC disease screening applications.	Lack of standardized sampling and analysis protocols leads to high variability and limited reproducibility in clinical conditions.	[43]

**Table 2:** Examples of major human breath biomarkers related to various diseases.

Exhaled VOCs	Potentially Diseases	Refs.
Formaldehyde, acetaldehyde, pentanal, hexanal, heptanal, octanal, nonanal, undecane	Lung cancer	[11,13,46,72,74]
Acetone	Non-alcoholic liver disease	[73]
Acetone	Diabetes	[46,75,76,77]
Acetophenone, formaldehyde, heptanal	Breast cancer	[78,79,80]
Acetaldehyde, acetone	COVID-19	[81,82]
Benzaldehyde	Chronic obstructive pulmonary disease	[83]
Tolualdehyde Malondialdehyde	Cystic fibrosis Oxidative stress and inflammation	[84,85,86]
Glutathione Malondialdehyde	Asthma	[87]
2-Heptanone 2-Nonanone 2-Undecanone	*Francisella tularensis* infection	[88]

**Table 3:** Reported concentrations of selected aldehydes and ketones in the exhaled breath of patients with LC and diabetes.

Compound	Disease	Concentration of VOCs in EB	Refs.
Acetone	T1D	4.9 ppm	[96]
Acetone	T2D	1.5 ppm	[96]
Nonanal	LC	44.0 pM	[97]
Octanal	LC	23.0 pM	[97]
Hexanal	LC	37.3 pM	[97]
Pentanal	LC	19.1 pM	[97]
Propanal	LC	53.6 pM	[97]

**Table 4:** Comparison of technologies for analysis of VOCs present in EB.

Technology	Advantages	Disadvantages
GC-MS	High sensitivity, specificity, and the ability to quantify compounds, and relatively fast analysis. The capability to detect compound mixtures.	Requires sample pre-concentration. High equipment and operating costs, along with complex operations requiring trained operators.
SIFT-MS	Provides high sensitivity. Enables real-time analysis of EB. There is no need for preconcentration of analytes.	Methods involve expensive instrumentation, technical complexity, and typically require skilled operators.
ENs	The ability to perform rapid and non-invasive analysis, enabling real-time detection without the need for complex sample preparation. Can be miniaturized and adapted for use in PoC. Easy to operate.	Characterized by a relatively low specificity towards individual chemical compounds, it can be significantly affected by various environmental factors, particularly fluctuations in humidity, which may interfere with its accuracy and reliability.
PTR-MS	Requires no sample preparation. Quick response time enables real-time detection of VOCs. Compact design allows for space-saving or portable configurations and simple operation. High resolution and sensitivity.	Difficulties in determining complex mixtures with undefined composition. The inability to detect compounds with an affinity for protons is lower than that of water. High costs associated with measurement equipment.
Raman Spectroscopy	Non-destructive, requires minimal sample preparation, and offers high specificity. Possibility of real-time analysis.	Limited sensitivity for some compounds. High equipment cost. Data interpretation complexity, especially in complex VOC mixtures.
Quantitative Nuclear Magnetic Resonance (qNMR)	Enables the determination of the compound’s structure as well as the quantification of VOCs.	Very expensive equipment requires skilled operators. Relatively low sensitivity compared to GC-MS. Due to the size of the device, it is unsuitable for use in PoC mode.
Chemiluminescence Detection	Offers high sensitivity and capability for specific detection.	Applicable only to chemiluminescent analytes, susceptible to interference, and may require optimized conditions.
Fluorescence Spectroscopy	High sensitivity and the capability to selectively detect specific compounds through the use of fluorophores.	Limited to VOCs exhibiting fluorescence properties. Background fluorescence may interfere with signal interpretation and often requires optimization of experimental conditions.

**Table 5:** Examples of commercial EN devices and employed transducer technologies.

EN Devices	Type of Transducer Technology	Refs.
BIONOTE (Bionote, Big Lake, MN, USA)	QCM sensors with anthocyanin-coated gold electrodes	[123,124]
SpiroNose (Breathomix, Leiden, The Netherlands)	Sensor arrays, each composed of MOS sensors	[123,125]
Aeonose (The eNose Company, Zutphen, The Netherlands)	Micro hotplate MOS	[123,126]
DiagNose (Figaro Engineering, Osaka, Japan)	MOS sensors	[127,128]
Cyranose 320 (Sensigent, Baldwin Park, CA, USA)	Carbon black–polymer composite chemiresistor	[123,128]
Owlstone Lonestar (Owlstone Medical, Cambridge, UK)	Field asymmetric ion mobility spectrometry	[123]

**Table 6:** Examples of recent MOS-based chemiresistors utilizing nanostructured recognition materials for the detection of biomarkers present in EB.

Material/Structure	Analyte	Sensor Response Range/LOD [ppm]	Working Temperature	Refs.
Au-modified ZnO nanofoam	Acetone	20–100/–	275 °C	[143]
In_2_O nanocube	Formaldehyde	–/25	225 °C	[144]
SnO_2_ nanosheet with nanoparticle + noble metal catalyst	Nonanal	1–10/–	300 °C	[145]
Au/SnO_2_	Nonanal	–/9.5	250 °C	[146]
SnO_2_ nanoparticles	Acetaldehyde	–/40	100 °C	[147]
Nano-SnO_2_ powders	Hexanal	–/0.05	200 °C	[148]

**Table 7:** Comparison of biosensors based on OBPPs and enzymes for the detection of aldehydes and ketones.

Biosensor	Method	Analytes	LOD [ppm]/ Sensor Response Range [ppm]	Refs.
OBPP1	QCM	Acetaldehyde Octanal	243/–	[164]
			455/–	
OBPP3	QCM	Acetaldehyde Octanal	571/–	[164]
			49/–	
OBPP4	QCM	Acetaldehyde	327/981–3988	[156]
		Hexanal	186/558–1826	
		Octanal	114/342–1437	
		Nonanal	14/42–1303	
OBPP4-GSGSGS	QCM	Nonanal	2/–	[157]
S-ADH/NADH	Fluorescence (fiber-optic)	Acetone	–/0.02–5.3	[163]
ADH/NADH (reverse reaction)	Fluorescence (fiber-optic)	Acetaldehyde	–/0.02–10	[165]
LEKKKKDC-NH_2_	QCM	Acetaldehyde	1/–	[154]
Aldehyde dehydrogenase	Fluorescence	Trans-2-nonenal	0.23/0.4–7.5	[166]

**Table 8:** Examples of gas sensors based on MIPs used for the detection of aldehydes and ketones. The table provides detailed information about the sensor types, target analytes, template molecules, functional monomers, and polymerization methods. The examples illustrate the diversity of MIP materials used in sensor technology.

Sensor	Analyte	Template	Functional Monomers	Polymerization Method	Refs.
MIP titanium dioxide nanotube array	Formaldehyde	Formaldehyde	Pyrrole	Electropolymerization	[173]
MIP-coated QCM	Formaldehyde	Formaldehyde	2-(Trifluoromethyl) acrylic acid, ethylene glycol dimethacrylate, 1-hydroxycyclohexyl phenyl ketone	Photopolymerization	[174]
MIP-coated QCM	Decanal	Decanal	Methacrylic acid, ethylene glycol dimethacrylate, 2,2′-azobis-isobutyronitrile	Free radical polymerization	[175]
MIP and AuNP chemiresistive sensor	Acetone	Acetone	Methyl methacrylate	Free radical polymerization	[176]
MIP interdigitated in gold electrodes	Acetaldehyde	Acetaldehyde	Pyrrole	Electropolymerization	[177]

**Table 9:** Comparison of MIP-based sensors for the detection of aldehydes and ketones.

Sensor	Analyte	LOD [ppm]	Sensor Response Range [ppm]	Refs.
MIP interdigitated in gold electrodes	Acetaldehyde	500	–	[177]
MIP-AuNPs	Nonanal	4.5	2.5–100	[178]
MIP-MWCNTs	Hexanal	10	10–200	[179]
MIP-AuNPs	Acetone	66	50–300	[176]
IP NPs	Formaldehyde	0.5	–	[180]

## Data Availability

No new data were created or analyzed in this study.

## Abbreviations

The following abbreviations are used in this manuscript:

AI	Artificial Intelligence
EB	Exhaled Breath
EN	Electronic Nose
FET	Field-Effect Transistor
GC-IMS	Gas Chromatography–Ion Mobility Spectrometry
GC-MS	Gas Chromatography–Mass Spectrometry
LC	Lung Cancer
LOD	Limit of Detection
MIPs	Molecularly Imprinted Polymers
ML	Machine Learning
MOS	Metal–Oxide Semiconductor
NPs	Nanoparticles
OBP	Odorant-Binding Protein
OBPP	Odorant-Binding Protein-Derived Peptide
PoC	Point-of-Care
PPB	Parts per Billion
PPM	Parts per Million
PTR-MS	Proton Transfer Reaction–Mass Spectrometry
QCM	Quartz Crystal Microbalance
SERS	Surface-Enhanced Raman Spectroscopy
SIFT-MS	Selected Ion Flow Tube–Mass Spectrometry
VOCs	Volatile Organic Compounds
